# HS-SPME-GC-MS Combined with Orthogonal Partial Least Squares Identification to Analyze the Effect of LPL on Yak Milk’s Flavor under Different Storage Temperatures and Times

**DOI:** 10.3390/foods13020342

**Published:** 2024-01-22

**Authors:** Jinliang Zhang, Liwen Zhong, Pengjie Wang, Juan Song, Chengrui Shi, Yiheng Li, William Oyom, Hao Zhang, Yanli Zhu, Pengcheng Wen

**Affiliations:** 1 College of Food Science and Engineering, Gansu Agricultural University, Lanzhou 730070, China; zjl1467638475@163.com (J.Z.); zliw1181@163.com (L.Z.); songjuan2113@163.com (J.S.); shicr0918@163.com (C.S.); yihenglixy@163.com (Y.L.); 2 College of Food Science and Nutritional Engineering, China Agricultural University, Beijing 100083, China; wpj1019@cau.edu.cn (P.W.); zhanghaocau@cau.edu.cn (H.Z.); 3 Food and Nutritional Sciences Program, North Carolina Agricultural and Technical State University, Greensboro, NC 27411, USA; woyom@aggies.ncat.edu; 4 China-Malaysia National Joint Laboratory, Biomedical Research Center, Northwest Minzu University, Lanzhou 730030, China

**Keywords:** yak milk, flavor substance, *Lipoprotein lipase*

## Abstract

Flavor is a crucial parameter for assessing the sensory quality of yak milk. However, there is limited information regarding the factors influencing its taste. In this study, the effects of endogenous lipoprotein lipase (LPL) on the volatile flavor components of yak milk under storage conditions of 4 °C, 18 °C and 65 °C were analyzed via headspace solid-phase microextraction gas chromatography-mass spectrometry (HS-SPME-GC-MS) combined with orthogonal partial least-squares (OPSL) discrimination, and the reasons for the changes in yak milk flavors were investigated. Combined with the difference in the changes in volatile flavor substance before and after the action of LPL, LPL was found to have a significant effect on the flavor of fresh yak milk. Fresh milk was best kept at 4 °C for 24 h and pasteurized for more than 24 h. Principal component analysis (PCA) and orthogonal partial least squares discriminant analysis (OPLS-DA) were employed to characterize the volatile components in yak milk under various treatment conditions. Twelve substances with significant influence on yak milk flavor were identified by measuring their VIP values. Notably, 2-nonanone, heptanal, and ethyl caprylate exhibited OAV values greater than 1, indicating their significant contribution to the flavor of yak milk. Conversely, 4-octanone and 2-heptanone displayed OAV values between 0.1 and 1, showing their important role in modifying the flavor of yak milk. These findings can serve as monitoring indicators for assessing the freshness of yak milk.

## 1. Introduction

Flavor is one of the important sensory indicators of milk and its dairy products which can affect consumer preferences [1]. Flavor compounds encompass a variety of chemical substances, including aldehydes, alcohols, ketones, hydrocarbons, esters, ethers, and furans, as well as nitrogen- and sulfur-containing compounds. They are affected by factors such as storage time, storage temperature, sterilization conditions, and the degree of lipolysis. 

The yak is a special breed of herbivorous cattle that has undergone long-term natural selection and self-adaptation [2]. Due to its extreme living environment, yak milk has very high levels of nutrients (fat, protein, minerals), and especially a milk fat content as high as 6–8%. The triglycerides in milk fat will be hydrolyzed by lipase to produce free fatty acid [3,4], which will be converted into more volatile flavor substances, thus giving yak milk a unique flavor that is different from that of ordinary cow’s milk. However, the dispersed location of grazing areas and the inconvenient transportation from production areas make it difficult to collect and transport fresh yak milk. Therefore, yak milk needs to be stored and transported for a long time before it reaches large-scale dairy production sites. During the transportation and storage of yak milk, the membrane of the milk fat globule ruptures under mechanical shear, and LPL enters the milk fat globule and combines with its internal triglycerides to produce free fatty acids, which are converted into ketones, alcohols, acids, aldehydes, and other volatile flavor substances, which can easily lead to the degradation of the flavor quality of yak milk [5,6]. Therefore, the study of the mechanism of the flavor deterioration of yak milk during transportation can provide a basis for delaying and inhibiting the flavor fission of yak milk during storage and transportation, and provide a guarantee for the development and utilization of high-quality yak milk products.

The combination of HS-SPME and GC-MS can enhance the sensitivity of the detection of volatile flavor compounds and has been widely used for determining volatile compounds in food [7]. Jia et al. [8] established and validated a mass spectrometry method for the analysis of flavor compounds in goat’s milk and cow’s milk, and identified and analyzed 42 flavor compounds; Chi et al. [9] used headspace solid-phase microextraction-gas chromatography -mass spectrometry (HS-SPME-GC-MS) and an electronic nose (E-nose) combined with multivariate statistical analysis to identify 62 flavor compounds from six skimmed milks. The volatile flavor components mainly include fatty acids, esters, ketones, hydrocarbons, aldehydes, lactones, etc. Liu et al. [10] studied the effect of Lactobacillus plantarum on the flavor characteristics of fermented walnut milk using HS-SPME-GC-MS. Contemporary research on yak milk flavor has focused on extrinsic conditions such as heat treatments, and even less on the effect of endogenous enzymes on its flavor. In this study, we investigated the differences in the effects of LPL on the flavor compounds of yak milk at different storage temperatures and times, using an HS-SPMEE-GC-MS technique combined with OPLS-DA (orthogonal partial least squares discriminant analysis) and OAV (Original Video Animation) analysis. The aim was to explore the influence of lipase on the flavor of yak milk under different storage conditions and provide a theoretical and technical basis for the regulation of yak milk’s flavor.

## 2. Materials and Methods

### 2.1. Experimental Design

Yak milk collected from 100 healthy yaks in Gashu Village, Luqu County, Gannan, Tibetan Autonomous Prefecture, was mixed and encapsulated in sterile jars within 2 h and stored at 4 °C for its rapid return to the laboratory.

The specific experimental steps of this study are as follows:

The samples were stored under different storage time and temperature conditions to monitor the changes in the LPL activity, TG (triglyceride) content, fatty acid content, and volatile flavor substance content of yak milk. The specific storage conditions were as follows: 4 °C storage (6 h, 18 h, 30 h), 18 °C storage (6 h, 18 h, 30 h), and 65 °C storage, with 30 min treatment (6 h, 18 h, 30 h); for convenience of analysis these were denoted as S- (6 h, 18 h, 30 h), M- (6 h, 18 h, 30 h), and H- (6 h, 18 h, 30 h). S denotes the 4 °C treatment, M denotes the 18 °C treatment, and H denotes the pasteurization treatment. This treatment entailed the addition of 0.5 g of purified LPL to 10 mL of milk before processing it at 65 °C for 30 min. It was stored at 18 °C for 30 h and its volatile aroma substances measured.

### 2.2. Analysis of TG Changes

The TG content was determined according to Li et al.’s [11] method and some modifications were made. The triglyceride content of yak milk was determined using a triglyceride content detection kit.

### 2.3. Fatty Acid Content Analysis

The operation was carried out following the national standard GB 5009.168-2016 [12]. The internal standard used was undecanoic acid (≥98%; Accela, San Ramon, CA, USA) with a concentration of 1 g/L. Qualitative and quantitative analyses of fatty acids were conducted.

### 2.4. LPL Isolation and Purification

According to the method of Wither et al. [13], affinity chromatography purification was used to separate and purify yak milk LPL at 4 °C. A total of 1 L of yak milk was centrifuged at 4000× *g* for 15 min to separate the fat, and skim milk was obtained by discarding the upper floating liquid. NaCl was added to the skim milk to reach a concentration of 350 mmol/L, and 25 mL of Heparin-Sepharose CL-6B was added to the skim milk and mixed at 4 °C with gentle stirring (200 r/min) for 12 h. Then, LPL-adsorbed Heparin-Sepharose CL-6B was separated via centrifugation at 6000× *g*, and the Heparin-Sepharose CL-6B adsorbed with LPL was packed into a chromatography column (20 mm × 100 mm). The column was washed with a large amount of barbitone sodium (5 mmol/L)-hydrochloric acid buffer solution (pH 7.4) containing NaCl (0.16 mol/L) until the eluate reached a stable absorbance at 280 nm. Then, LPL was eluted with a gradient of barbitone sodium (5 mmol/L)-hydrochloric acid buffer solution (pH 7.4) containing 0.5–2.0 mol/L NaCl. The enzymatically active fractions were collected and combined, and dialysis was performed using barbitone sodium (5 mmol/L)-hydrochloric acid buffer solution (pH 7.4) containing 0.15 mol/L NaCl. These were stored through freeze-drying or storage at −30 °C.

### 2.5. LPL Enzyme Activity Assay

LPL activity in yak milk was determined using a Lipase Assay Kit III as described previously Zhang et al. [14].

### 2.6. LPL Protein Content Analysis

The method proposed by Jayasena et al. [15], with slight modification, was used to determine LPL protein content using an ELISA assay kit.

### 2.7. LPL Molecular Weight Measurement

According to the method described by Kwiatkowska-Semrau et al. [16], discontinuous urea SDS-PAGE (Sodium Dodecyl Sulfate Polyacrylamide Gelelectrophoresis) electrophoresis was used to determine the molecular weight of endogenous LPL in yak milk. The separating gel concentration was 15%, and the stacking gel concentration was 5%. After diluting LPL with loading buffer, 10 µL of the sample was loaded into the sample well. The electrophoresis chamber was operated at a stable voltage of 120 V, and after entering the separating gel, the voltage was stabilized at 80 V. The separated proteins were stained with Coomassie Brilliant Blue for 1 h, and then destained with destaining solution (acetic acid: methanol: water = 7.5:7.5:85, *v*:*v*:*v*). Finally, the gel was imaged and the molecular weight of LPL was determined by comparing it with the molecular weight of standard proteins and using gel imaging software (BIO-RAD) based on the migration rate of standard proteins.

### 2.8. HS-SPME-GC-MS Analysis

Take yak milk to measure its volatile aroma substances. Add 0.5 g of purified LPL to 10 mL of milk and process it at 65 °C for 30 min. Store it at 18 °C for 30 h and measure its volatile aroma substances. Make slight modifications based on the method of Fang et al. [17].

HS-SPME conditions: 10 mL of milk sample, 2 g of sodium chloride, and 1 μL of internal standard solution (2-methyl-3-heptanone) with a concentration of 0.816 μg/μL were placed in a headspace sample bottle equipped with a magnetic stirrer (20 mL). The bottle was heated in a constant-temperature water bath at 40 °C for 30 min. A manual HS-SPME injector was fixed on the SPME mounting device, and the extraction needle was inserted into the headspace bottle, with the extraction fiber extending to approximately 5 mm above the surface of the milk. The headspace was adsorbed for 30 min. After adsorption, the extraction fiber was quickly inserted into a GC/MS injector for desorption for 5 min.

GC conditions: SE-54 capillary column (30 m × 0.25 mm × 0.25 μm), injector temperature 250 °C; temperature program, initial temperature 30 °C, hold for 1 min, ramp at a rate of 5 °C/min to 250 °C, no hold; helium was used as the carrier gas with a flow rate of 1 mL/min, no split injection.

MS conditions: EI ion source, electron energy 70 eV; transfer line temperature 250 °C; ion source temperature 230 °C; quadrupole temperature 150 °C; mass scan range 50 u–380 u; scan mode, full scan; solvent delay 5 min.

Qualitative analysis: The volatile substances detected by MS were matched with the NIST 11 spectral library for retrieval, and the retention index (RI) was calculated. Components with a retention match greater than 600 were considered. The calculation method for the RI values is shown in the formula

$$RI=100n-100\times \frac{t_{r}-t_{n}}{t_{n+1}-t_{n}}$$

In this formula, *RI* is the retention index; *n* and *n* + 1 are the numbers of carbon atoms in the straight-chain alkanes before and after the unknown substance elutes; $t_{n+1}$ and tn are the retention times of the straight-chain alkanes; and *t_r_* is the retention time of the unknown substance in the gas chromatography (*t_n_* < *t_r_* < *t_n_*_+1_).

Quantitative analysis: 2-methyl-3-heptanone is used as an internal standard, with a relative factor of one for each compound. Based on the mass concentration of the internal standard, the peak area of each component in the sample, and the peak area of the internal standard, calculate the content of each component in the yak milk sample. The semi-quantitative analysis formula for each component is

$$m_{i}=\frac{m_{0}\times V_{0}\times {\;A}_{i}}{V_{i}\times A_{0}}$$

In this formula, *m_i_* represents the mass concentration of the unknown substance, in μg/L; *m*_0_ represents the mass concentration of the internal standard, in μg/μL; *A_i_* represents the peak area of the unknown substance; *A*_0_ represents the peak area of the internal standard; *V_i_* represents the volume of the milk sample added during extraction, in L; *V*_0_ represents the volume of the internal standard solution added, in μL.

### 2.9. Sensory Evaluation

Referring to the method of ISO 11035 [18], with slight modifications, and according to GB/T 16291.1-2012 [19]. “Sensory Analysis—general guidance for the selection, training and monitoring of assessors”, sensory evaluators who were healthy and free from a tobacco or alcohol habit were selected from the laboratory of the School of Food Science and Engineering of Gansu Agricultural University to conduct sensory analysis. A total of 20 sensory evaluators (10 males and 10 females) were selected to form a sensory panel. Subsequently, the panelists received sensory training according to the consensus method described by Skelton et al. [20]. The training was held five times a week, for two hours each time, and lasted for one month. Informed consent was obtained from all participants.

A total of 27 milk samples were obtained, and all yak milk samples were numbered (M1–M27). To help to understand the effect of different storage temperatures and time on the flavor of yak milk, the samples obtained were classified into low-temperature storage (LM_6_, LM_18_, LM_30_), average-pasture-temperature storage (AM_6_, AM_18_, AM_30_), and pasteurized storage (PM_6_, PM_18_, PM_30_). Each yak milk sample (15 mL) used for sensory testing was stored in a 50 mL glass bottle in a suitable and constant-temperature (25 °C) laboratory environment. Yak milk samples were drawn and evaluated by each evaluator.

Odor descriptors based on 2-nonanone (sweet aroma), heptanal (fat waxy flavor), ethyl ocaprylate (sweetness), 4-octanone (nutty flavor), and 2-heptanone (milky flavor) were evaluated, with scores ranging across a 10-point scale, as shown in Table 1 (0–1, none; 2–3, weak; 4–5, slightly weak; 6–7, average; 8–9, slightly strong; and 10, strong). The sensory scores of the 20 panelists’ averages were plotted on a spiderweb graph.

### 2.10. Data Processing

The data were represented as mean ± standard deviation (SD). A three-way ANOVA was conducted in order to investigate the effect of yak milk pasteurization, storage time, and storage temperature on flavor, and Tukey’s multiple comparison test was used to determine significant differences (*p* < 0.05). Statistical analyses were performed using SPSS 20.0. Multivariate analysis was conducted using Origin 2021 software.

The reliability of the results was identified using PCA plots made in SIMCA14.1, and the relationship between the fatty acid content and volatile flavor compounds of yak milk was evaluated using partial least squares regression (PLSR) analysis, and substances with VIP (Variable Importance in Projection) scores greater than 1 were screened using OPLS-DA to determine their contribution to yak milk flavor. All experiments were performed in triplicate.

## 3. Results

### 3.1. LPL Analytical Purification and Characterization

**Figure 1:**
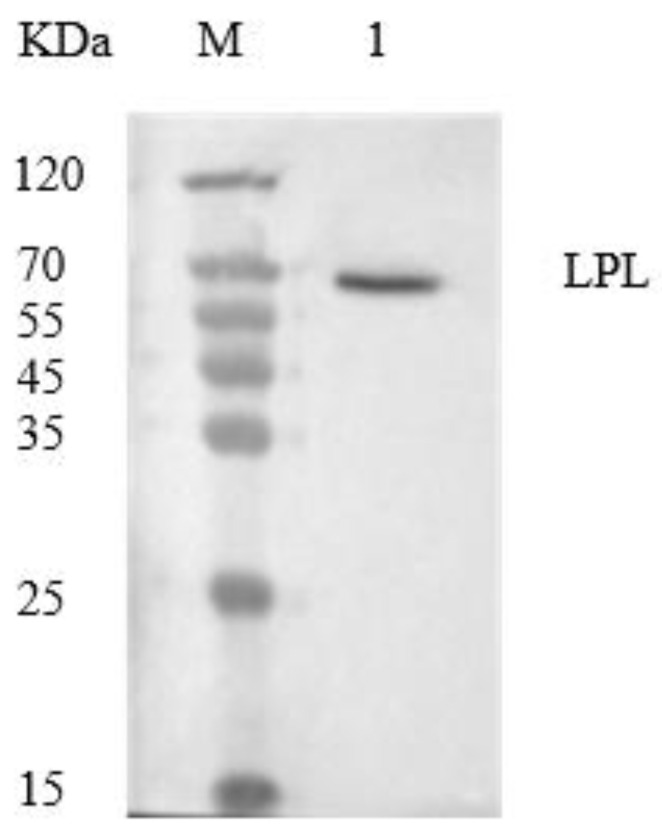
LPL gel electrophoresis (M: Maker; 1: LPL).

The enzyme activity of LPL can reflect the conversion rate of the substrate. Table 2 indicates that after purification by heparin Sepharose CL-6B, the specific activity of LPL is 4113.6 U/g, its purification factor is 3344, and its recovery rate is 30%. The high-enzyme-activity components were combined, concentrated, and separated by SDS-PAGE (15% separation gel, 5% concentration gel) electrophoresis. The separation results are shown in Figure 1. According to the migration rate of standard proteins and LPL in SDS-PAGE, analyzed by gel imaging software, the molecular weight of LPL is 65 KDa. After separation by SDS-PAGE, LPL appears as a single protein band, indicating a high purity of the purified LPL. The molecular weight of yak milk LPL is consistent with the reported molecular weight of purified LPL from milk by Egelrud and Olivecrona [21], which is 62~66 KDa.

### 3.2. Analysis of Changes in LPL Activity in Yak Milk under Different Storage Conditions

**Figure 2:**
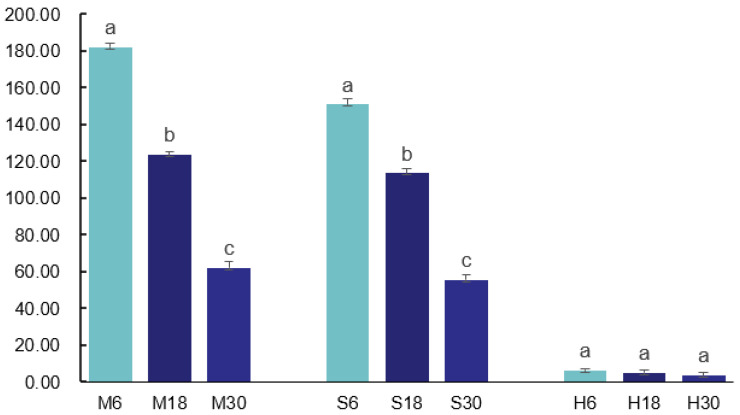
LPL activity in yak milk under different treatments. Bars indicate standard error (±SE). Different letters represent significant differences within groups (*p* < 0.05). S6, S18, S30: storage at 4 °C for 6, 18, and 30 h; M6, M18, M30: storage at 18 °C for 6, 18, and 30 h; H6, H18, H30: pasteurization treatment before storage for 6, 18, and 30 h.

In this study, changes in LPL activity were observed by treating and storing yak milk under different conditions. The results are shown in Figure 2, with an increase in storage time, the LPL activity decreased more significantly (*p* < 0.05) in the M and S groups. LPL activity was extremely low and did not change significantly in the H treatment group, which indicated that the LPL basically dissipated its activity after the pasteurization treatment. LPL activity was higher in the S and M treatment groups, and LPL activity in the M treatment group was higher than that in S treatment group, while LPL activity was diminished in the pasteurization treatment group.

### 3.3. Analysis of the Effect of LPL on TG in Yak Milk

**Figure 3:**
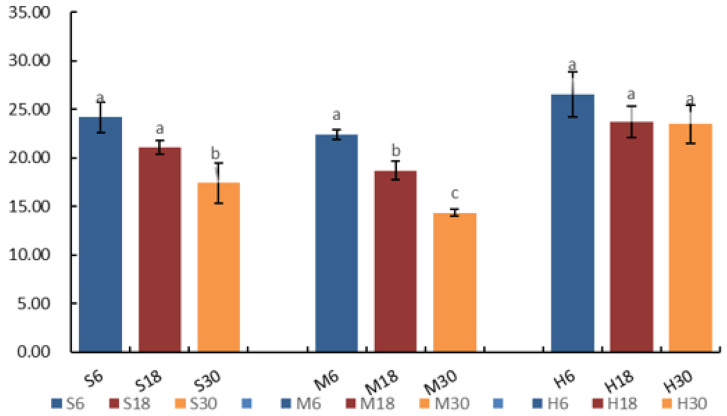
The residual amount of triglyceride in yak milk under different treatments. Bars indicate standard error (±SE). Different letters represent significant differences within groups (*p* < 0.05). S6, S18, S30: storage at 4 °C for 6, 18, and 30 h; M6, M18, M30: storage at 18 °C for 6, 18, and 30 h; H6, H18, H30: pasteurization treatment before storage for 6, 18, or 30 h.

In this study, changes in the TG decomposition rate by LPL were observed in yak milk under different storage and processing conditions. The results showed that, as seen in Figure 3, the TG content gradually decreased with the increase in storage time. The TG content of the M30 group was 14.36 mg/mL, and the value of the TG content of the H6 group was 26.55 mg/mL, whereas the TG content decreased from 26.55 to 23.46 mg/mL in H6 compared to H30, a decrease of 11.64%. At 46 mg/mL, the change in TG content was not significant (*p* > 0.05), indicating that the activity of LPL was basically dissipated after the pasteurization treatment, and thus the rate of TG decomposition was lower. The TG content of the S6 and S30 groups decreased from 24.19 mg/mL to 17.42 mg/mL, a decrease of 27.99%, with a significant change in TG (*p* < 0.05), suggesting that the treatment at 4 °C resulted in a higher rate of TG decomposition due to the higher activity of LPL. The TG content of M6 and M30 decreased from 22.41 mg/mL to 14.36 mg/mL, a decrease of 35.92%, with a significant (*p* < 0.05) change in TG; this is the highest activity of LPL indicated, and therefore the highest rate of TG decomposition was observed when yak milk was stored at 18 °C. The TG content was higher in the pasteurized treatment group than in the other treatment groups, and the rate of decrease was higher in the M treatment group than in the S treatment group.

### 3.4. Effect of LPL on the Fatty Acid Content in Yak Milk

This study used GC-MS to analyze the changes in the fatty acid content of yak milk under different storage temperatures and times, as shown in Table 3. In the S treatment group, the content of caproic acid (C6:0), caprylic acid (C8:0), and capric acid (C10:0) significantly increased with storage time (*p* < 0.05), while the content of myristic acid (C14:0), palmitic acid (C16:0), and heptadecanoic acid (C17:0) significantly decreased (*p* < 0.05). In the H treatment group, there were significant changes in the content of capric acid (C10:0) and myristic acid (C14:0). In the M treatment group, the content of caproic acid (C6:0), caprylic acid (C8:0), capric acid (C10:0), and lauric acid (C12:0) significantly increased, while the content of other fatty acids significantly decreased. This suggests that as LPL activity increases, the short-chain fatty acid content also increases, while long-chain fatty acid content decreases. Palmitic acid (C16:0), stearic acid (C18:0), and myristic acid (C14:0) were the main saturated fatty acids with a higher content.

### 3.5. Effect of LPL on the Characteristic Flavor Substances of Yak Milk

**Figure 4:**
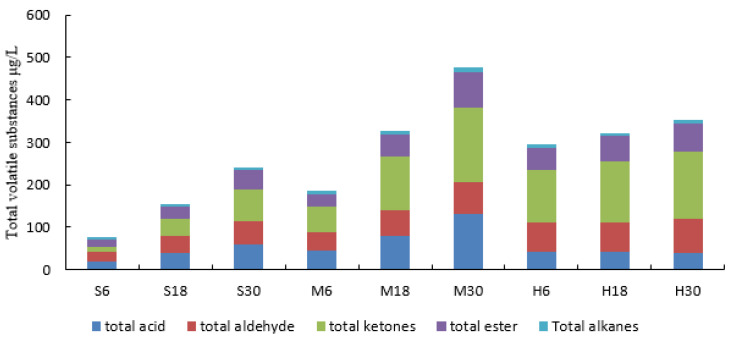
Residual amount of triglyceride in yak milk under different treatments. Bars indicate standard error (±SE). (S6, S18, S30: storage at 4 °C for 6, 18, and 30 h; M6, M18, M30: storage at 18 °C for 6, 18, and 30 h; H6, H18, H30: pasteurization treatment before storage for 6, 18, and 30 h).

**Figure 5:**
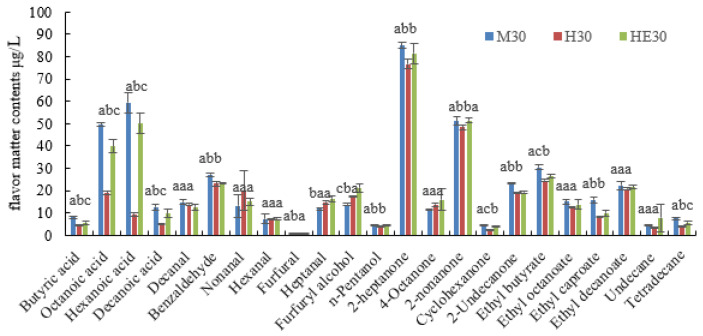
Volatile flavor substances in yak milk after adding LPL. Storage at 18 °C for 30 h (M30), pasteurized before storage for 30 h (H30), pasteurized milk with LPL added for 30 h (HE30). The different lowercase letters represent significant differences between the groups (*p* < 0.05).

The volatile components of yak milk samples treated with different storage temperatures and times are shown in Table 4 and Figure 4. A total of 23 volatile compounds were identified in the yak milk including four fatty acids, two alcohols, six aldehydes, two alkanes, four esters, and five ketones. With the increase in storage time, the volatile content increased by 300.75 μg/L from M6 to M30, 168.95 μg/L from S6 to S30, and 62.77 μg/L from H6 to H30. The total volume of volatiles in M was higher than that in both H and S and the trend of the increase was more rapid. The impact of LPL on the volatile flavor substances in yak milk is shown in Figure 5. The total volume of volatile substances in the HE 30 group is higher than that in the H treatment group and lower than that in the M treatment group. The changes in the acidic substances are significant (*p* < 0.05), as well as those in the benzaldehyde and heptanal (*p* < 0.05), and ketone substances (*p* < 0.05). The initial volatile content of the H treatment group was higher than that of the S treatment group, but the increase was low. The LPL activity of the M treatment group was higher than that of the S treatment group, indicating that the higher the LPL activity, the higher the content of volatile flavor substances produced, while in the pasteurization treatment LPL acted rapidly with TG to produce volatile substances during the warming process, so the initial content of the H treatment group was high, while the LPL activity was basically dispersed after the completion of the pasteurization, so the increase in its volatile content was low.

### 3.6. Correlation between Fatty Acid Composition and the Volatile Components of Yak Milk

**Figure 6:**
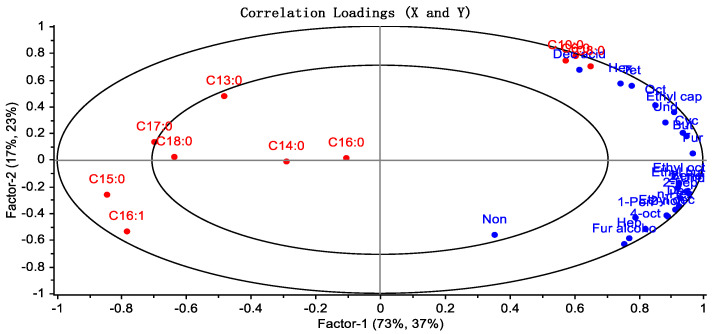
Total kinds of volatile flavor substances. (S6, S18, S30: storage at 4 °C for 6, 18, and 30 h; M6, M18, M30: storage at 18 °C for 6, 18, and 30 h; H6, H18, H30: pasteurization treatment before storage for 6, 18, and 30 h).

PLSR analysis was used to evaluate the relationship between fatty acids and volatile compounds [22]. The results, as shown in Figure 6, indicated a positive correlation between the levels of C6:0, C8:0, and C10:0 and the content of the acidic substances heptanal, hexyl acetate, furfural, and cyclohexanone. Acids play an important role in the formation of flavor in yak milk. They not only serve as flavor substances but also act as precursors for ketones, alcohols, esters, and other flavor components [23]. Aldehydes can quickly be reduced to ethanol or oxidized to acids [24]. Enzymatic hydrolysis can rapidly generate small amounts of fresh and acidic compounds, but with a lower intensity of taste. Therefore, fatty acids have an impact on the volatile flavor of yak milk.

### 3.7. Flavor Contribution Analysis

**Figure 7:**
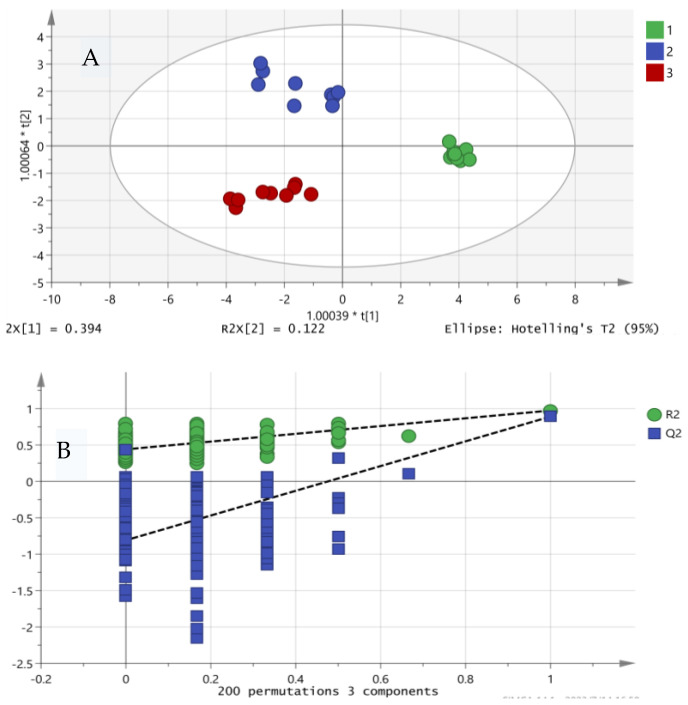
Arrangement of volatile flavor compounds after storage under different conditions; PCA score plot (**A**) and 200 permutation tests (**B**). In (**A**), green indicates 4 °C treatment, blue indicates 18 °C treatment, and red indicates pasteurization treatment.

The results obtained from the PCA analysis of different yak milk treatments are shown in Figure 7A. Distinct separations of various degrees were displayed in the nine milk samples, with a total 51.6% contribution to the variation of PC1 and PC2. The large between-group and small within-group differences for the different temperature treatments indicate that temperature has a significant effect on the lipolytic capacity of LPL, whereas the small within-group difference at 4 °C suggests that perhaps the lipolytic effect of LPL does not change significantly with increasing time at 4 °C. This is in agreement with the results of the experiments, which demonstrated the effectiveness of PCA for differentiating between the various skim milk samples.

Additionally, models were established using PCA and OPLS-DA to classify volatile flavor compounds in differently treated yak milks, and the classification performance of the models was good. The substances that contribute significantly to the separation were screened using OPLS-DA. The fitting parameters R2 X(cum), R2 Y(cum), and Q2 (cum) of the model were 0.952, 0.965, and 0.863, respectively, indicating the good fitness and predictability of the OPLS-DA model. The stability of the model was validated through 200 permutation tests, and the results are shown in Figure 7B. The intercept values of R2 and Q2 were 0.274 and −0.786, respectively, indicating that the OPLS-DA model in this study is stable and not overfitting.

**Figure 8:**
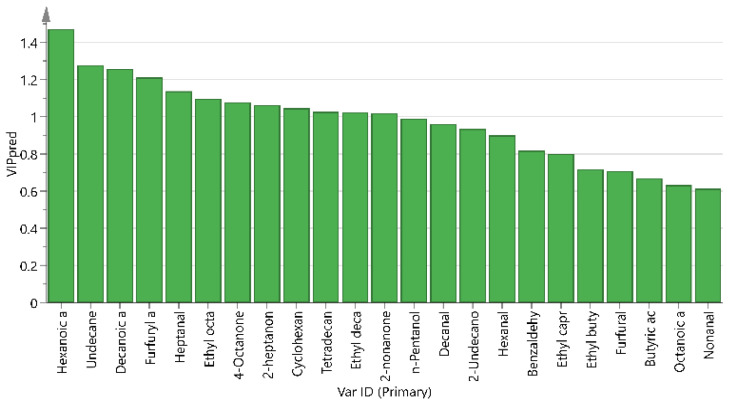
VIP values of volatile compounds in yak milk across different treatments.

In Figure 8, it can be seen that twelve volatile flavor substances with VIP scores > 1 were screened across different treatments, including hexanoic acid, undecanoic acid, decanoic acid, furfuryl alcohol, heptanal, ethyl octanoate, 4-octanone, 2-heptanone, cyclohexanone, tetradecane, ethyl caprylate, and 2-nonanone. The aroma characteristics of yak milk cannot be judged solely on the content of the aroma components; however, the aroma components with higher OAV values can be used to indicate the aroma characteristics of yak milk. As shown in Table 5, according to the olfactory threshold value in the existing literature [25], the OAV values obtained by calculating the ratio between the content of the aroma substances and the olfactory threshold value, an OAV > 1, demonstrated that the aroma component had a certain influence on the aroma of yak milk. 2-Nonanone, heptanal, and ethyl octanoate had an OAV > 1, which was considered to indicate that these three substances contributed more to the flavor of yak milk, while 4-octanone and 2-heptanone had an OAV < 1; specifically, their OAVs were in the range of 0.1–1, so it was concluded that these two substances played an important role in modifying the flavor of yak milk.

### 3.8. Sensory Evaluation

**Figure 9:**
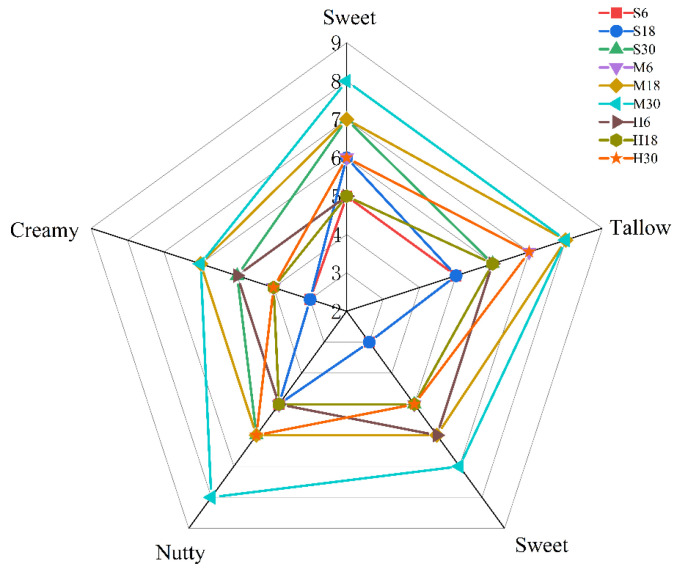
Radar chart of volatile substances that had significant effects on the flavor of yak milk. S6, S18, S30: storage at 4 °C for 6, 18, and 30 h; M6, M18, M30: storage at 18 °C for 6, 18, and 30 h; H6, H18, H30: pasteurization treatment before storage for 6, 18, and 30 h).

The sensory evaluation results were judged in terms of sweet aroma, fat waxy flavor, sweetness, nutty flavor, and milky flavor. According to the data shown in Figure 9, M30 scored the highest, which indicates that the highest flavor intensity is generated under the treatment condition of 18 °C for 30 min. In contrast, the flavor intensity of the 4 °C treatment group was slightly lower than that of the 65 °C treatment group. Therefore, refrigerated transportation at 4 °C should be preferred during storage and transportation.

## 4. Discussion

The aroma of yak milk is a significant sensory evaluation parameter, which can influence its level of acceptance among consumers. In comparison to regular cow milk, yak milk possesses a higher milk fat content and a larger milk fat globule size, leading to a more delicate and sensitive milk fat globule membrane [26,27]. Consequently, the triglycerides enclosed within the milk fat globule membrane are more susceptible to degradation, resulting in the production of free fatty acids that impact the flavor of yak milk. In this article, we investigated the effects of storage temperature and storage time on the triglycerides in yak milk, and the results showed that the TG residue was the smallest in the group treated at 18 °C for 30 h, while the TG residue was the most substantial in the group treated at 65 °C for 30 min, and the change in TG residue was not significant over time. Temperature can have an effect on the activity of LPL [28], while the degree and rate of triglyceride hydrolysis depends on the activity of LPL [29]. At 18 °C, the activity of LPL was higher and TG was more easily broken down, while when treated at 65 °C for 30 min, the activity of LPL was almost nothing, so more TG remained. The 4 °C treatment inhibited enzyme activity, thus reducing the decomposing rate of TG. Therefore, different temperatures affect enzyme activity, leading to different amounts of TG decomposition, which in turn produces different amounts of fatty acids.

Yak milk has a high content of palmitic, stearic, and myristic acids. With the increase in time in the 18 °C treatment group, the content of medium- and short-chain fatty acids increased significantly, while, in the 4 °C treatment group and 65 °C for 30 min treatment group, the increase in medium- and short-chain fatty acids’ contents was not significant, which was consistent with the results for residual TG. This indicated that different temperature and time treatments affected enzyme activity, which led to different hydrolysis rates of TG by LPL and consequently different fatty acid contents. Free fatty acids can be converted into substances such as methyl ketones, esters, lactones, alcohols, and aldehydes, which directly affect the flavor of yak milk [30]. Therefore, it is necessary to identify the volatile flavor substances in yak milk treated at different temperatures and for different lengths of time. The experimental results showed that the content of volatile flavor substances was the highest in the 18 °C treatment group, while the content was relatively low in the 4 °C treatment group and the 65 °C for 30 min treatment group. At 18 °C LPL activity was high, and the decomposition rate of TG was fast, such that more fatty acids were produced and then converted into volatile flavor substances. Their content in the 65 °C for 30 min treatment group was relatively low because the temperature of the milk gradually increased during the heating process, and the activity of LPL was enhanced. Thus, the hydrolysis rate of TG was accelerated within a certain period of time, which led to an increase in the volume of flavor substances, but the increase in volatile substances was not significant because of the heat inactivation of LPL when it reached 65 °C. The activity of LPL was inhibited at 4 °C, and the volume of volatile substances produced was lower than that of the treatment at 65 °C, but, because the activity of LPL was not dispersed, the volume of volatile substances increased faster than that of the treatment at 65 °C with the extension of time. Therefore, fresh milk is best stored at 4 °C within 24 h, and after more than 24 h fresh milk is best stored following pasteurization.

According to the PLSR model graph, it can be seen that the contents of C6:0, C8:0, and C10:0 are positively correlated with the content of the acidic substances nonanal, hexanoic acid ethyl ester, furfural, and cyclohexanone. This indicates that C6:0, C8:0, and C10:0 may be flavor precursors of these substances. The group treated at 65 °C had lower levels of short-chain fatty acids and volatile acid compounds, indicating that pasteurization has a greater impact on flavor. Pasteurization has a significant effect on the activity of microorganisms and enzymes in milk. Short-chain fatty acids are catalyzed by enzymes to form other flavor substances under the action of microorganisms [31]. Microorganisms and exogenous lipoprotein lipases also have an impact on the flavor of yak milk. Other members of our team are conducting specialized research on microorganisms and exogenous lipoprotein lipases, so this article does not discuss the impact of microorganisms and exogenous lipoprotein lipases on the flavor of yak milk. Combining the results of TG content and fatty acid content, it can be concluded that the esterification reaction catalyzed by lipases has a significant enhancing effect on volatile substances.

The volatile flavor substances’ content does not fully represent the extent of their contribution to flavor, and the flavor of milk is determined by both the volatile substances’ content and the threshold value. The OAV refers to the ratio of the aroma concentration to its threshold value, and it is generally accepted that compounds with an OAV ≥ 1 contribute more to the flavor of fermented milk, and compounds with 0.1 ≤ OAV < 1 play an important role in modifying the flavor of milk [32]. The results of the OPLS-DA and VIP screening of different treatment groups showed that hexanoic acid, undecanoic acid, decanoic acid, furfuryl alcohol, heptanal, ethyl octanoate, 4-octanone, 2-heptanone, cyclohexanone, tetradecane, ethyl decanoate, and 2-nonanone were the volatile flavor substances that contributed more to the flavor. Combining the results of the OAV calculations for these twelve compounds, an OAV > 1 was found for 2-nonanone, heptanal, and ethyl octanoate. These are considered to be the main source of yak milk flavor, while the OAV of 4-octanone and 2-heptanone was 0.1 < OAV < 1, which means they are considered to be an important modifier of the flavor of yak milk. Ketones are produced by the β-oxidation of saturated fatty acid, degradation of amino acids, or microbial metabolic reactions, and these substances have obvious flavor characteristics and low flavor thresholds, whereas 2-heptanone and 2-nonanone generate the milky and sweet flavors of yak milk, which contribute more to the typical flavor of yak milk. Aldehydes are produced by the oxidation of milk fat. Due to their low flavor threshold, aldehydes have a typical fat aroma at low concentrations, but concentrations above the threshold produce rancid, sour, or other odors [33]. The content of aldehydes in yak milk is high and continues to increase with time, while heptanal presents a pungent fat-wax odor, resembling a fruity odor, and according to the sensory evaluation chart the fat-wax odor was the strongest after storage at 18 °C for 30 h. Therefore, it is believed that heptanal has a greater contribution to the undesirable flavors of yak milk. Esters are formed by the esterification of alcohols and acyl-CoA generated through various metabolic pathways, which have a strong aromatic odor, and ethyl caprylate has a better effect on the flavor of yak milk, as it generates a fruity aroma. In conclusion, 2-heptanone, 2-nonanone, 4-octanone, and ethyl octanoate had a good effect on the flavor of yak milk, while heptanal added a bad flavor to yak milk. In this study, the correlation models established by PCA, clustered heat map, Venn diagram, and PLSR comprehensively explored the correlation between the implications of different flavor characteristics, and effectively revealed realistic differences in the volatile flavor traits of yak milk samples after different treatments.

## 5. Conclusions

This study investigated the aroma compounds in yak milk using GC-MS in combination with orthogonal partial least squares discriminant analysis. Through the comprehensive evaluation of an aroma intensity analysis, sensory orientation, TG residue, and fatty acid content, it was determined that LPL had a strong influence on the flavor of yak milk. The identified typical volatile compounds were 2-nonanone, heptanal, ethyl octanoate, 4-octanone, and 2-heptanone. These compounds are important volatile flavor substances formed through the conversion of fatty acids by the action of LPL on TG. Particularly, 2-nonanone, heptanal, and ethyl octanoate were found to be significant compounds related to the odor of yak milk, through OAV analysis. Based on the results obtained from GC-MS, as well as OPLS-DA and VIP analysis, employing low-temperature transportation for short-distance transport and a pasteurization treatment for long-distance transport is recommended. These measures will help extend the shelf life of yak milk and ensure its quality during transportation to large-scale dairy factories and consumers.

## Figures and Tables

**Table 1:** Sensory evaluation scoring sheet.

Fragrant Substance	Sensory Description	Score
2-Nonanone	sweet aroma	(0–1, none; 2–3, weak; 4–5, slightly weak; 6–7, average; 8–9, slightly strong; 10, strong)
heptanal	fat waxy flavor	(0–1, none; 2–3, weak; 4–5, slightly weak; 6–7, average; 8–9, slightly strong; 10, strong)
Ethyl octanoate	sweetness	(0–1, none; 2–3, weak; 4–5, slightly weak; 6–7, average; 8–9, slightly strong; 10, strong)
4-Octanone	nutty flavor	(0–1, none; 2–3, weak; 4–5, slightly weak; 6–7, average; 8–9, slightly strong; 10, strong)
2-Heptanone	milky flavor	(0–1, none; 2–3, weak; 4–5, slightly weak; 6–7, average; 8–9, slightly strong; 10, strong)

**Table 2:** The purification of LPL from yak milk.

	Volume	Enzyme Activity (U/L)	Total Activity (U)	Protein Concentrations (g/L)	Activity (U/g)	Recovery Rate	Purification Fold
Yak milk	1000 mL	62	48	50.21	1.23	100	1
Purified LPL	300 mL	181	54.3	0.044	4113.6	30	3344

**Table 3:** Saturated fatty acid content of yak milk under different treatments.

Fatty Acid (g/100 g)	S6	S18	S30	M6	M18	M30	H6	H18	H30
C6:0	0.66 ± 0.08 ^b^	1.43 ± 0.34 ^a^	1.91 ± 0.45 a	1.49 ± 0.56 ^b^	2.34 ± 0.34 ^b^	3.31 ± 0.40 ^a^	0.62 ± 0.01 ^a^	0.70 ± 0.10 ^a^	0.72 ± 0.03 ^a^
C8:0	0.44 ± 0.11 ^b^	0.76 ± 0.08 ^a^	0.76 ± 0.03 a	0.85 ± 0.06 ^b^	1.41 ± 0.38 ^a,b^	1.90 ± 0.40 ^a^	0.44 ± 0.06 ^a^	0.46 ± 0.03 ^a^	0.50 ± 0.05 ^a^
C10:0	1.33 ± 0.23 ^b^	2.42 ± 0.43 ^b^	3.06 ± 0.22 a	2.98 ± 0.10 ^b^	3.41 ± 0.62 ^b^	4.21 ± 0.17 ^a^	1.58 ± 0.06 ^b^	1.68 ± 0.05 ^a^	1.72 ± 0.03 ^a^
C12:0	4.39 ± 1.69 ^a^	2.96 ± 0.26 ^a^	2.95 ± 0.03 ^a^	2.65 ± 0.06 ^b^	2.69 ± 0.21 ^b^	3.14 ± 0.11 ^a^	2.33 ± 0.09 ^a^	2.54 ± 0.10 ^a^	2.57 ± 0.16 ^a^
C13:0	0.06 ± 0.07 ^a^	0.05 ± 0.08 ^a^	0.03 ± 0.06 ^a^	0.07 ± 0.12 ^a^	0.00 ± 0.00 ^a^	0.00 ± 0.00 ^a^	0.00 ± 0.00 ^a^	0.07 ± 0.07 ^a^	0.00 ± 0.00 ^a^
C14:0	7.02 ± 0.26 ^a^	6.73 ± 0.29 ^a^	4.49 ± 0.40 ^b^	6.07 ± 0.44 ^a^	5.03 ± 0.35 ^b^	3.69 ± 0.18 ^c^	4.87 ± 0.29 ^b^	5.88 ± 0.96 ^a^	5.90 ± 0.55 ^a^
C15:0	0.06 ± 0.10 ^a^	0.67 ± 0.10 ^b^	0.23 ± 0.20 ^b^	0.59 ± 0.06 ^a^	0.25 ± 0.08 ^b^	0.24 ± 0.21 ^b^	0.00 ± 0.00 ^a^	0.92 ± 0.80 ^a^	0.02 ± 0.03 ^a^
C16:0	18.83 ± 1.16 ^a^	16.84 ± 0.27 ^b^	15.79 ± 0.55 ^b^	17.92 ± 0.31 ^a^	15.93 ± 0.31 ^b^	12.69 ± 0.58 ^c^	17.21 ± 1.03 ^a^	18.54 ± 0.52 ^a^	16.95 ± 1.71 ^a^
C16:1	0.00 ± 0.00 ^b^	0.54 ± 0.02 ^a^	0.00 ± 0.00 ^b^	0.49 ± 0.05 ^a^	0.11 ± 0.18 ^b^	0.21 ± 0.19 ^a,b^	0.00 ± 0.00 ^a^	0.60 ± 0.52 ^a^	0.01 ± 0.02 ^a^
C17:0	1.83 ± 1.23 ^a^	0.24 ± 0.22 ^b^	0.00 ± 0.00 ^b^	0.19 ± 0.06 ^a^	0.00 ± 0.00 ^b^	0.02 ± 0.03 ^b^	0.1 ± 0.18 ^a^	0.19 ± 0.16 ^a^	0.00 ± 0.00 ^a^
C18:0	5.70 ± 0.63 ^a^	5.48 ± 0.28 ^a^	5.51 ± 0.72 ^a^	6.45 ± 0.18 ^a^	5.53 ± 0.21 ^b^	4.96 ± 0.63 ^b^	5.76 ± 1.86 ^a^	5.36 ± 0.60 ^a^	5.18 ± 0.71 ^a^

Storage at 4 °C for 6, 18, and 30 h (S6, S18, S30). Storage at 18 °C for 6, 18, and 30 h (M6, M18, M30). Pasteurization treatment before storage for 6, 18, and 30 h (H6, H18, H30). In the same group and across different columns, The different lowercase letters represent significant differences between the groups (*p* < 0.05).

**Table 4:** Volatile substance content of differently treated milks.

Name of Compound	RI	Content (μg/L)								
		S6	S18	S30	M6	M18	M30	H6	H18	H30
Butyric acid	775	2.06 ± 0.36 ^c^	3.49 ± 0.13 ^a^	6.37 ± 0.40 ^a^	3.82 ± 0.26 ^c^	5.59 ± 0.25 ^a^	8.21 ± 0.56 ^a^	5.22 ± 0.10 ^a^	5.1 ± 0.09 ^a^	4.87 ± 0.13 ^a^
Octanoic acid	1005	10.4 ± 1.18 ^c^	16.38 ± 2.23 ^a^	28.94 ± 2.31 ^a^	10.46 ± 2.14 ^c^	22.55 ± 4.41 ^a^	49.57 ± 0.89 ^a^	20.13 ± 0.72 ^a^	19.55 ± 0.12 ^a^	18.94 ± 0.70 ^a^
Hexanoic acid	984	4.47 ± 0.15 ^c^	9.64 ± 1.57 ^a^	14.61 ± 0.99 ^a^	21.29 ± 1.61 ^c^	40.52 ± 3.23 ^a^	59.4 ± 4.54 ^a^	11.6 ± 2.42 ^a^	11.03 ± 0.99 ^a^	9.43 ± 0.78 ^a^
Decanoic acid	1365	3.16 ± 0.74 ^a^	8.71 ± 1.15 ^a^	10.2 ± 1.42 ^a^	8.1 ± 0.34 ^c^	10.2 ± 0.77 ^a^	12.67 ± 1.28 ^a^	6.3 ± 0.35 ^a^	5.74 ± 0.33 ^a^	5.19 ± 0.15 ^a^
Total acid		20.10	38.22	60.13	43.67	78.86	129.85	43.26	41.43	38.44
Decanal	1204	3.05 ± 0.14 ^c^	4.98 ± 0.21 ^a^	10.31 ± 0.89 ^a^	7.44 ± 0.33 ^c^	11.07 ± 0.55 ^a^	14.85 ± 1.21 ^a^	11.96 ± 0.71 ^a^	13.32 ± 0.39 ^a^	13.91 ± 0.54 ^a^
Benzaldehyde	982	5.1 ± 0.40 ^c^	11.72 ± 0.70 ^a^	15.91 ± 0.62 ^a^	7.99 ± 0.56 ^c^	18.14 ± 0.8 ^a^	27.09 ± 0.64 ^a^	19.39 ± 0.81 ^a^	20.15 ± 0.44 ^a^	23.13 ± 0.95 ^a^
Nonanal	1104	6.42 ± 0.75 ^a^	14.22 ± 0.43 ^a^	15.43 ± 1.52 ^a^	19.47 ± 6.89 ^a^	14.47 ± 3.09 ^a^	13.22 ± 5.12 ^a^	18.22 ± 8.72 ^a^	16.63 ± 4.27 ^a^	20.16 ± 9.03 ^a^
Hexanal	806	3.94 ± 0.29 ^c^	4.74 ± 0.04 ^a^	6.16 ± 0.51 ^a^	5.35 ± 0.30 ^a^	6.46 ± 0.16 ^a^	7.45 ± 2.35 ^a^	6.87 ± 0.12 ^a^	7.07 ± 0.05 ^a^	7.24 ± 0.16 ^a^
Furfural	1463	0.34 ± 0.01 ^c^	0.57 ± 0.05 ^a^	0.84 ± 0.06 ^a^	0.57 ± 0.02 ^c^	0.81 ± 0.02 ^a^	1.15 ± 0.10 ^a^	0.65 ± 0.03 ^c^	0.80 ± 0.02 ^a^	0.90 ± 0.04 ^a^
Heptanal	905	2.44 ± 0.23 ^a^	5.21 ± 2.13 ^a^	5.24 ± 0.72 ^a^	3.34 ± 0.78 ^c^	9.03 ± 0.88 ^a^	11.78 ± 0.76 ^a^	11.1 ± 1.39 ^a^	12.41 ± 0.49 ^a^	14.82 ± 0.73 ^a^
Total aldehyde		21.29	41.44	53.88	44.17	59.97	75.55	68.20	70.38	80.16
Furfuryl alcohol	861	4.27 ± 0.13 ^a^	4.73 ± 0.08 ^a^	7.31 ± 0.61 ^a^	8.22 ± 0.62 ^c^	11.37 ± 0.50 ^a^	13.77 ± 0.63 ^a^	14.06 ± 0.37 ^c^	15.16 ± 0.56 ^a^	17.39 ± 0.36 ^a^
1-Pentanol	761	0.72 ± 0.03 ^c^	1.51 ± 0.02 ^a^	1.67 ± 0.13 ^a^	0.63 ± 0.17 ^c^	1.26 ± 0.12 ^a^	4.58 ± 0.17 ^a^	3.17 ± 0.31 ^c^	3.61 ± 0.16 ^a^	4.16 ± 0.05 ^a^
Total alcohol		4.98	6.24	8.98	8.85	12.64	18.35	17.235	18.77	21.56
Cyclohexanone	891	0.63 ± 0.02 ^c^	0.88 ± 0.04 ^a^	1.62 ± 0.16 ^a^	1.15 ± 0.06 ^c^	3.22 ± 0.52 ^a^	4.61 ± 0.30 ^a^	2.03 ± 0.08 ^c^	2.06 ± 0.05 ^a^	2.33 ± 0.10 ^a^
2-heptanone	853	3.06 ± 0.38 ^c^	15.49 ± 1.30 ^a^	31.3 ± 4.22 ^a^	31.57 ± 2.63 ^c^	66.77 ± 2.00 ^a^	85.08 ± 1.19 ^a^	53.21 ± 2.17 ^c^	65.32 ± 2.70 ^a^	76.7 ± 2.11 ^a^
4-Octanone	1937	2.29 ± 0.18 ^c^	3.16 ± 0.44 ^b^	8.69 ± 0.10 ^a^	7.25 ± 0.23 ^c^	9.43 ± 0.31 ^b^	11.33 ± 0.31 ^a^	10.5 ± 0.69 ^c^	11.98 ± 0.57 ^b^	13.79 ± 0.88 ^a^
2-nonanone	1052	3.73 ± 0.43 ^c^	11.27 ± 2.05 ^b^	21.68 ± 1.06 ^a^	10.92 ± 3.07 ^c^	31.51 ± 0.67 ^b^	51.22 ± 1.9 ^a^	39.66 ± 0.69 ^c^	45.03 ± 1 ^b^	48.37 ± 1.00 ^a^
2-Undecanone	1294	3.43 ± 0.47 ^c^	8.70 ± 1.10 ^b^	10.87 ± 1.42 ^a^	9.23 ± 2.80 ^c^	15.87 ± 0.86 ^b^	23.39 ± 0.44 ^a^	16.6 ± 0.32 ^b^	17.74 ± 1.18 ^a,b^	18.89 ± 0.13 ^a^
Total ketones		13.14	39.49	74.16	60.13	126.81	175.63	121.99	142.13	160.07
Ethyl butyrate	785	8.16 ± 1.43 ^c^	14.36 ± 1.31 ^b^	18.26 ± 0.64 ^a^	9.67 ± 0.61 ^c^	16.51 ± 3.18 ^b^	30.37 ± 1.02 ^a^	20.2 ± 1.08 ^c^	22.66 ± 0.63 ^b^	24.63 ± 0.42 ^a^
Ethyl octanoate	1220	2.03 ± 0.85 ^b^	3.32 ± 1.27 ^b^	8.12 ± 0.32 ^a^	9.32 ± 1.29 ^c^	12.44 ± 1.34 ^b^	15.03 ± 1.17 ^a^	9.87 ± 1.30	11.22 ± 0.96	12.57 ± 0.43
Ethyl caproate	984	4.32 ± 0.32 ^b^	5.97 ± 2.45 ^b^	11.67 ± 1.14 ^a^	6.15 ± 0.66 ^c^	10.93 ± 1.33 ^b^	15.99 ± 1.32 ^a^	8.22 ± 0.10 ^b^	8.35 ± 0.06 ^a,b^	8.45 ± 0.08 ^a^
Ethyl decanoate	1391	1.55 ± 0.32 ^c^	4.51 ± 0.68 ^b^	7.25 ± 0.69 ^a^	5.09 ± 1.60 ^c^	12.99 ± 0.51 ^b^	22.32 ± 1.92 ^a^	15.99 ± 0.41 ^c^	18.54 ± 0.56 ^b^	20.88 ± 0.40 ^a^
Total esters		16.05	28.16	45.30	30.23	52.87	83.70	54.28	60.78	66.54
Undecane	1150	2.6 ± 0.14 ^c^	3.19 ± 0.12 ^b^	3.65 ± 0.14 ^a^	3.7 ± 0.08 ^c^	4.25 ± 0.03 ^b^	4.55 ± 0.07 ^a^	3.36 ± 0.01 ^c^	3.51 ± 0.08 ^b^	3.68 ± 0.05 ^a^
Tetradecane	1483	3.52 ± 0.18 ^b^	3.99 ± 0.28 ^a,b^	4.54 ± 0.46 ^a^	3.71 ± 0.19 ^c^	4.87 ± 0.31 ^b^	7.58 ± 0.42 ^a^	3.44 ± 0.17 ^b^	3.85 ± 0.25 ^a^	4.09 ± 0.07 ^a^
Total alkanes		6.12	7.18	8.19	7.42	9.12	12.13	6.80	7.35	7.77
Total		81.68	160.72	250.63	194.46	340.26	495.21	311.77	340.85	374.54

Data are shown as mean ± standard deviation (n = 3); values with different letters in a row indicate a significant difference (*p* < 0.05) based on one-way ANOVA analysis using Duncan multiple range tests. Storage at 4 °C for 6, 18, and 30 h (S6, S18, S30). Storage at 18 °C for 6, 18, and 30 h (M6, M18, M30). Pasteurization treatment before storage for 6, 18, and 30 h (H6, H18, H30). In the same group and across different columns, the different lowercase letters represent significant differences between the groups (*p* < 0.05).

**Table 5:** Different aroma components’ OAV from different yak milk treatments.

Name of Compound	Threshold Value (μg/L)	OAV								
		S6	S18	S30	M6	M18	M30	H6	H18	H30
Hexanoic acid	2517	0.002	0.004	0.006	0.008	0.016	0.024	0.005	0.004	0.004
Decanoic acid	3000	0.001	0.003	0.003	0.003	0.003	0.004	0.002	0.002	0.002
Furfuryl alcohol	2000	0.002	0.002	0.004	0.004	0.006	0.007	0.007	0.008	0.009
Heptanal	3	0.812	1.737	1.746	1.113	3.010	3.928	3.701	4.138	4.941
Ethyl octanoate	12	0.158	0.258	0.631	0.724	0.967	1.168	0.767	0.872	0.977
4-Octanone	100	0.023	0.032	0.087	0.073	0.094	0.113	0.105	0.120	0.138
2-heptanone	140	0.022	0.111	0.224	0.226	0.477	0.608	0.380	0.467	0.548
Cyclohexanone	240	0.003	0.004	0.007	0.005	0.013	0.019	0.008	0.009	0.010
Ethyl decanoate	1122	0.001	0.004	0.006	0.005	0.012	0.020	0.014	0.017	0.019
2-nonanone	5	0.745	2.253	4.336	2.185	6.303	10.244	7.932	9.006	9.673

Data are shown as mean ± standard deviation (n = 3); Storage at 4 °C for 6, 18, and 30 h (S6, S18, S30). Storage at 18 °C for 6, 18, and 30 h (M6, M18, M30). Pasteurization treatment before storage for 6, 18, and 30 h (H6, H18, H30). Threshold value: olfactory threshold in water.

## Data Availability

Data is contained within the article.
